# Differing endometrial expression of calcium modulating transient receptor potential channels

**DOI:** 10.1186/s12967-021-02763-z

**Published:** 2021-03-17

**Authors:** Maryam Ghavideldarestani, Alexandra E. Butler, Stephen L. Atkin

**Affiliations:** 1 Hull York Medical School, Cottingham Road, Hull, HU67RX UK; 2 Diabetes Research Center (DRC), Qatar Foundation (QF), Biomedical Research Institute (QBRI), Hamad Bin Khalifa University (HBKU), Doha, Qatar; 3 Royal College of Surgeons Ireland, Bahrain, Adliya, Bahrain

To the editor

Assisted reproductive techniques (ART) have increased the live birth success rate, but there is still a significant implantation failure rate. Epithelial cell calcium homeostasis is tightly regulated by mechanisms that include activation of the TRP channel superfamily of 9 families including TRPC (Canonical) [1]. TRPCs are located on the cell membrane and act as receptor-operated channels (ROC), whilst cytosolic localization of these channels indicates their role as store-operated channels (SOC): both ROCs and SOCs are key players in the regulation of intracellular calcium homeostasis [2]. TRPC 1–4 and 6 expression in the bovine reproductive tract has been reported; these receptors exhibit hormone modulation [2] and calcium dysregulation can lead to menstrual disturbances [3], suggesting that TRPC receptors may modulate calcium in the human endometrium and affect implantation and fertility.

Endometrial samples at day 21 of the menstrual cycle (MC) from 15 patients (Table 1) prior to ART were taken; five women with normal female investigation, but with male factor infertility (MFI), acted as controls; five women had unexplained infertility (UI) and five had PCOS with no MFI. All patients in the UI and MFI groups had regular ovulatory menstrual cycles (a 28-day cycle ± 1-day), with no intermenstrual or postcoital bleeding or dysmenorrhoea. The diagnosis of PCOS was based on all three diagnostic criteria of the Rotterdam consensus [4], but endometrial samples were only taken from those sequential PCOS patients who had ovulated in that cycle as confirmed by the presence of a corpus luteum on ultrasound examination. None of the patients had reported successful pregnancy or miscarriage. All patients gave written consent and study approval was granted by the NHS UK Local Research Ethics.

**Table 1:** Demographic and biochemical data for the women with unexplained infertility, male factor infertility and polycystic ovary syndrome (PCOS) included in the study

	Unexplained infertility (n = 5)	Male factor infertility (n = 5)	PCOS (n = 5)
Age (years)	35.6 (2.7)	33.8 (4.0)	30.8 (2.3)
BMI (kg/m^2^)	26.8 (3.9)	26.7 (1.2)	26.3 (5.1)
AMH (ng/ml)	19.3 (14.0)	19.0 (9.9)	64.3 (8.7)***
Fasting glucose (nmol/l)	4.7 (0.3)	4.9 (0.5)	5.0 (0.2)
Insulin (IU/ml)	6.0 (0.6)	9.2 (3.0)	7.2 (6.9)
HOMA-IR	1.3 (0.2)	2.1 (0.8)	3.1 (3.2)
FAI	1.0 (0.0)	1.5 (0.6)	6.8 (4.3)*
CRP (mg/l)	1.6 (1.2)	2.3 (1.5)	3.4 (5.1)

Data are shown as mean ± standard deviation (SD)

*BMI* body mass index, *AMH* Anti-mullerian hormone, *HOMA-IR* Homeostatic Model Assessment of Insulin Resistance, *FAI* Free Androgen Index, *CRP* C-reactive protein

*p < 0.01, ***p < 0.0001

Total RNA was extracted using a NucleoSpin RNA II isolation kit (Macherey–Nagel, Germany), was reverse-transcribed to cDNA using an EZ-First Strand cDNA Synthesis Kit (Geneflow, Israel) and real-time PCR undertaken using using β-actin as the housekeeping gene [5]. Relative gene expression was analyzed using StepOne software V2.0 (Fisher Scientific, Loughborough, UK) and the baseline and threshold were set manually. RT-PCR data were analysed using the ΔΔCt method. Primer efficiency was determined with serial dilutions of the cDNA templates to generate a standard curve and the measurements fitted the linear range of the reaction (6). Direct sequencing of purified products was undertaken. Each experiment was performed using 5 samples in each group. One-way ANOVA was performed to test between MFI, UI and PCOS groups with a post hoc Tukey’s difference test. All statistical analysis was performed using Origin 6.1 software (OriginLab Corporation, Northampton, Massachusetts).

The only difference between the women with UI, MFI and PCOS was an elevated AMH and FAI in the PCOS women (p < 0.0001 and p < 0.01, respectively), Table 1. Seven TRPC channel isoforms were investigated and TRPC 1, TRCP 6 and TRPC 7 genes were expressed in all 15 endometrial tissue samples, whilst there was no expression of TRPC 2, 3, 4 and 5. Expression of epithelial Cytokeratin-18 was positive and vimentin negative, confirming specimen epithelial cell origin. TRPC 1 was upregulated 6.8-fold in the endometrium of UI, compared to patients with MFI and patients with PCOS (p = 0.009). TRPC 6 was upregulated in both UI and PCOS (2.2 and 5.6-fold, respectively, p = 0.01). TRPC 7 expression was decreased in UI patients and increased in patients with PCOS (0.1 and 9.8-fold, respectively, p = 0.001 and 0.04) compared to MFI who were used as the control group (Fig. 1a, b).

**Fig. 1:**
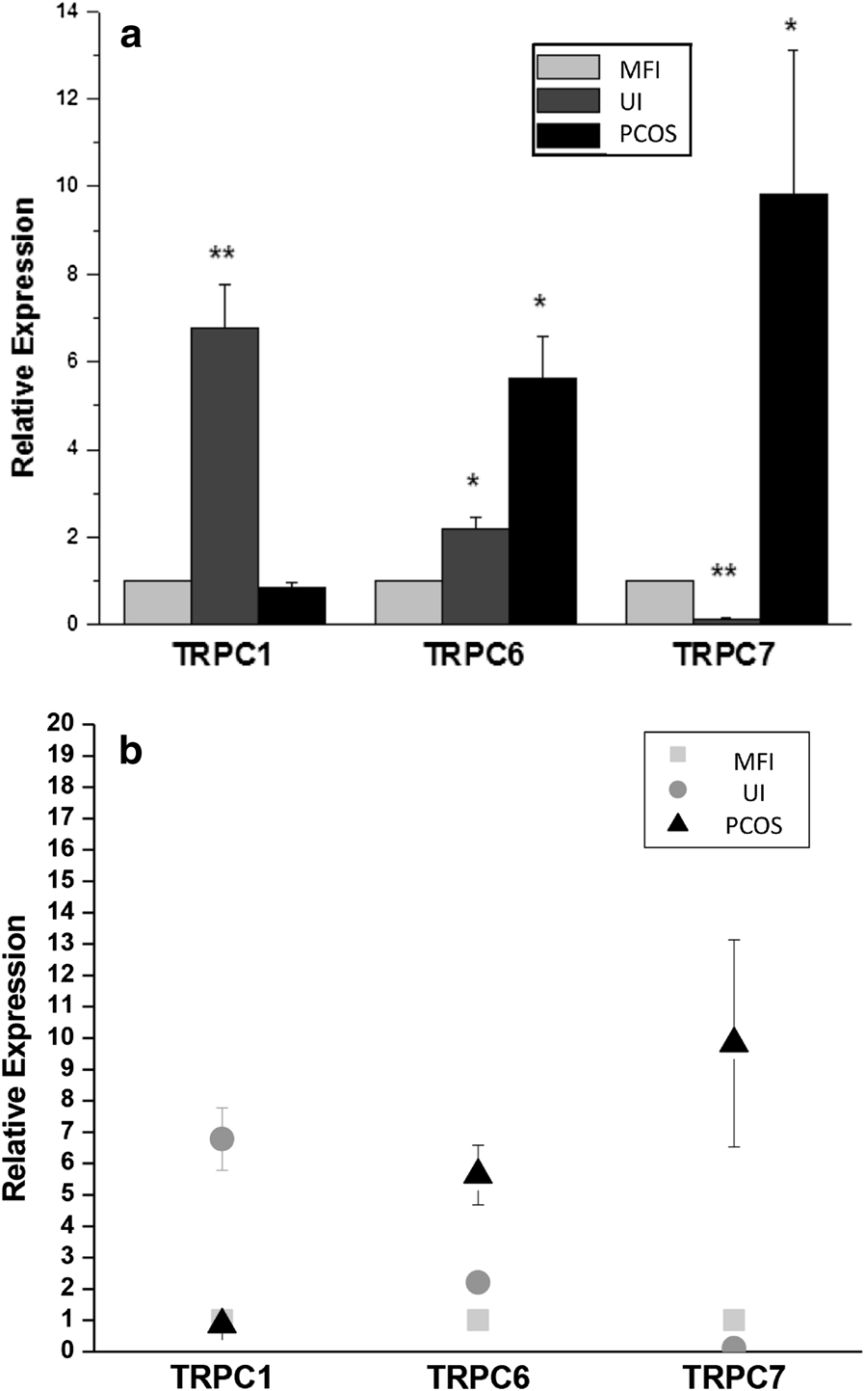
**a** Relative expression of TRPC 1, 6 and 7 by Real-Time PCR. TRPC 1 was up-regulated 6.8-fold in the endometrium of unexplained infertility (UI), compared to patients with male factor infertility (MFI) and patients with polycystic ovary syndrome (PCOS) (p = 0.009). TRPC 6 was upregulated in both UI and PCOS (2.2 and 5.6-fold, respectively, p = 0.01). TRPC 7 expression was decreased in UI patients and increased in patients with PCOS (0.1 and 9.8-fold, respectively, p = 0.001 and 0.04) compared to MFI who were used as the control group. **b** Scatter plot of the individual values shown in **a**

This study shows that TRPC 1, 6 and 7 were ubiquitously expressed in the endometrium that differed in the endometrium of women with differing causes of infertility. This is the first study on TRPC channels in patients with different causes of infertility biopsied at day 21 of the MC following ovulation. The MFI group were used as the reference group and showed significant differences in gene expression of TRPC 1, 6 and 7 between MFI and UI, and between MFI and PCOS. This suggests that endometrial factors, and possibly calcium flux-related mechanisms, could be the underlying problem in the UI group. Anovulation is the primary cause of infertility in PCOS: in this study all had ovulated and showed up-regulation of TRPC 6 and 7 gene expression compared to the MFI patients, a finding in accord with data that PCOS endometrium differs to normal women [7]. The data for TRPC 1 and TRPC 6 is in accord with that reported in whole endometrial biopsies [8] and that the TRPC channels are functional within endometrial stromal cells [8, 9]. Functional expression of TRPC 6 and TRPC 7 have recently been reported in human endometrial stromal cells [9]. These data are important, as it suggests that calcium modulation may have utility in fertility treatment with local therapeutic approaches.

A strength of this study is that all of the endometrial samples for mRNA were taken at day 21, the period of optimum implantation; however, the study is limited by the small population and future functional studies are needed to determine the role of calcium modulation in fertility.

In conclusion, human endometrium ubiquitously expresses TRPC 1, 6 and 7 on day 21 of the MC and the levels may differ depending on the cause of infertility in IVF patients, suggesting that modified calcium flux may be implicated in infertility.

## Data Availability

All data are available by contacting Dr Ghavideldarestani: maryam.ghavideldarestani@gmail.com.

## References

[1] Baracat MC, Serafini PC, Simoes Rdos S, Maciel GA, Soares JM, Baracat EC (2015). Systematic review of cell adhesion molecules and estrogen receptor expression in the endometrium of patients with polycystic ovary syndrome. Int J Gynaecol Obstet.

[2] Ghavideldarestani M, Atkin SL, Leese HJ, Sturmey RG. Expression and function of transient receptor potential channels in the female bovine reproductive tract. Theriogenology. 2016. 10.1016/j.theriogenology.2016.02.005 27001231

[3] Thys-Jacobs S, McMahon D, Bilezikian JP (2007). Cyclical changes in calcium metabolism across the menstrual cycle in women with premenstrual dysphoric disorder. J Clin Endocrinol Metab.

[4] Revised 2003 consensus on diagnostic criteria and long-term health risks related to polycystic ovary syndrome (PCOS). Hum Reprod. 2004;19(1):41–7. 10.1093/humrep/deh098 14688154

[5] Steinau M, Rajeevan MS, Unger ER (2006). DNA and RNA references for qRT-PCR assays in exfoliated cervical cells. J Mol Diagn.

[6] Bookout AL, Mangelsdorf DJ (2003). Quantitative real-time PCR protocol for analysis of nuclear receptor signaling pathways. Nucl Recept Signal.

[7] Piltonen TT, Chen JC, Khatun M, Kangasniemi M, Liakka A, Spitzer T (2015). Endometrial stromal fibroblasts from women with polycystic ovary syndrome have impaired progesterone-mediated decidualization, aberrant cytokine profiles and promote enhanced immune cell migration in vitro. Hum Reprod.

[8] Persoons E, Hennes A, De Clercq K, Van Bree R, Vriens G, O DF, et al. Functional Expression of TRP Ion Channels in Endometrial Stromal Cells of Endometriosis Patients. International journal of molecular sciences. 2018;19(9). 10.3390/ijms19092467 PMC6163224 30134548

[9] De Clercq K, Held K, Van Bree R, Meuleman C, Peeraer K, Tomassetti C (2015). Functional expression of transient receptor potential channels in human endometrial stromal cells during the luteal phase of the menstrual cycle. Hum Reprod.

